# The Impact of Cancer on Mental Health and the Importance of Supportive Services

**DOI:** 10.31661/gmj.v13i.3327

**Published:** 2024-02-26

**Authors:** Zhila Fereidouni, Samaneh Dehghan Abnavi, Zeinab Ghanbari, Roqayeh Gashmard, Fatemeh Zarepour, Neda Khalili Samani, Abraham Rajesh Sharma, Afsaneh Ghasemi

**Affiliations:** ^1^ Department of Nursing, School of Nursing, Fasa University of Medical Sciences, Fasa, Iran; ^2^ Department of Operating Room, Community-Oriented Nursing Midwifery Research Center, Nursing and Midwifery School, Shahrekord University of Medical Sciences, Shahrekord, Iran; ^3^ Department of Nursing, School of Nursing and Midwifery, Isfahan University of Medical Sciences, Isfahan, Iran; ^4^ Department of Nursing, Faculty of Nursing and Midwifery, Bushehr University of Medical Sciences, Bushehr, Iran; ^5^ Department of Nursing, School of Nursing and Midwifery, Ahvaz Jundishapur University of Medical Sciences, Ahvaz, Iran; ^6^ Department of Community Medicine, BJ Government Medical College, Pune, Maharashtra, India; ^7^ Department of Public Health, School of Health, Fasa University of Medical Sciences, Fasa, Iran

**Keywords:** Cancer, Mental Health, Suportive Services, Caregiver, Depression, Anaxity

## Abstract

Cancer is a complex disease that affects the physical and psychological well-being of the patient, their families, and caregivers. Indeed, cancer-related mental health disorders could impact treatment adherence, quality of life, and overall health outcomes. In addition, approximately 30% of patients may experience cancer-related psychological disorders, including anxiety, depression, and post-traumatic stress. Also, caregivers of patients with cancer can experience significant emotional, physical, and financial stress, which can have a negative impact on their health. Therefore, to address these issues, mental health resources should be integrated into cancer care settings to identify and intervene early for individuals with psychological distress. Hence, providing psychological support, counseling, and education about coping strategies could create a safe and supportive environment where individuals can express their emotions, reducing feelings of isolation and depression. However, there are some important barriers to accessing mental health support for individuals with cancer, including stigma, cultural attitudes, and financial and logistical challenges. Hence, strategies to overcome these barriers include increasing awareness and education about the importance of mental health care, providing integrated care that addresses both physical and mental health needs, and utilizing telehealth services. So, healthcare providers should continue to develop and implement innovative approaches to mental health care that are tailored to the essential requirements of individuals with cancer and to enhance knowledge regarding the key roles of mental health care for individuals with cancer.

## Introduction

Cancer is a significant global health issue and multifaceted disease that affects physical well-being and has profound psychological implications [1]. In addition, the psychological impact of cancer extends beyond the patient to their families and caregivers [2]. Cancer diagnosis often triggers various emotional responses, including fear, anxiety, and uncertainty about the future [3]. Facing treatment challenges, potential side effects, and the possibility of recurrence can further exacerbate these psychological burdens. Indeed, individuals with cancer may experience feelings of sadness, anger, grief, and a loss of control over their lives [4].

Studies indicated that neglecting mental health concerns can have far-reaching consequences, affecting treatment adherence, quality of life (QoL), and overall health outcomes [5][6]. Hence, mental health support plays a crucial role in enhancing coping mechanisms, promoting adherence to treatment protocols, and improving overall psychological well-being [5].

Integrating mental health resources into cancer care settings facilitates early identification and intervention for individuals with psychological distress [7]. Offering psychological support, counseling, and education about coping strategies could provide a safe and supportive environment where individuals can express their emotions openly and without judgment, which can alleviate feelings of isolation and depression [8][9].

Hence, the current review aimed to recognize and address the psychological impacts of cancer, and provide comprehensive and patient-centered care that acknowledges the multidimensional nature of the cancer.

## Prevalence and Patterns of Cancer-Related Mental Health Issues

The prevalence of cancer-related mental health issues varies depending on factors such as the type of cancer, stage of the disease, treatment modalities, and individual characteristics. However, research indicates that up to one-third of cancer patients experience symptoms of anxiety and depression during their treatment procedures [10].

The patterns of cancer-related mental health issues can manifest differently across individuals. Some common mental health challenges include heightened levels of anxiety, depression, post-traumatic stress disorder (PTSD), and adjustment disorders [11][12][13]. Anxiety often stems from the uncertainty surrounding the disease, treatment outcomes, and fear of recurrence [14]. Depression may be triggered by the emotional and physical upheaval caused by cancer, including symptoms such as fatigue, pain, and changes in body image [15]. PTSD can develop in individuals who have undergone traumatic medical procedures or experienced life-threatening cancer-related situations [16]. Adjustment disorders may arise as individuals struggle to cope with the emotional and lifestyle changes brought about by the diagnosis and treatment [17].

Certain subgroups of cancer patients are more vulnerable to mental health issues. For example, younger individuals with advanced disease stages, prior history of mental health conditions, and limited social support networks may be at higher risk [18][19]. Hence, it is crucial to understand and address the patterns of cancer-related mental health issues to provide appropriate support and intervention. Regular screening for distress and mental health symptoms can facilitate early identification and timely intervention [20]. Collaborative care models that involve oncology teams, mental health professionals, and support services can enhance coordination and ensure that patients receive comprehensive care [21].

## Psychological Effects of Cancer

Anxiety and Fear of Cancer Diagnosis, Treatment, and Prognosis

Cancer diagnosis can trigger intense anxiety and fear in individuals [11][14]. The initial shock and uncertainty surrounding the diagnosis can lead to a cascade of emotions, including worry about treatment options, prognosis, and the impact on one’s life [22].

One of the primary sources of anxiety is the fear of the treatment process itself [23]. Cancer treatments (such as surgery, chemotherapy, radiation therapy, and immunotherapy) are often associated with physical discomfort, side effects, and potential complications. Also, the fear of pain, loss of control, and the potential impact on their body image can increase anxiety levels [24].

Another source of anxiety is the uncertainty of the prognosis. Indeed, cancer is a complex disease, and outcomes could vary widely depending on factors such as the stage of cancer, type of cancer, treatment effectiveness, and individual response [25]. The fear of the unknown future, the possibility of recurrence, or disease progression can create a constant sense of stress and discomfort [26]. Therefore, the fear of cancer can extend to a broader existential fear as individuals are faced with their mortality and potential loss of life. On the other side, its impact on relationships, family, and future plans can become overwhelming, leading to increased anxiety levels [27]. Additionally, the fear and stigma associated with cancer within society may contribute to feelings of isolation and distress [28].

Depression and Mood Disorders Among Patients With Cancer

Depression among patients with cancer is more than just sadness; it involves persistent feelings of hopelessness, loss of interest in activities that were once pleasurable, changes in appetite or weight, sleep disturbances, fatigue, difficulty concentrating, and thoughts of death and/or suicide [12][29].

Various factors can influence cancer-related depression. In addition to fear associated with the diagnosis and treatment process, disruption in daily life, financial pressures, and impact on relationships contribute to emotional distress [30]. Additionally, the physical symptoms of cancer (e.g., pain and fatigue) can further exacerbate depressive symptoms [31].

PTSD and Cancer-Related Trauma

Experiencing cancer and its treatments can be traumatic for individuals, and it is not uncommon for patients with cancer to develop PTSD [32]. In other words, in the context of cancer, the trauma may stem from the diagnosis, invasive treatments, prolonged hospitalization, or the fear of recurrence [33]. Also, the physical pain, uncomfortable symptoms, and side effects of treatment may leave a lasting impact on individuals. Additionally, the fear of death, uncertainty about the future, and the loss of control over one’s health can contribute to the development of PTSD symptoms [34].

PTSD may present as nightmares, flashbacks, avoidance of cancer-related reminders or discussions, emotional numbness, hyperarousal, and difficulty concentrating or sleeping [35]. These symptoms can significantly impact a person’s QoL, daily functioning, and overall well-being.

## Psychosocial Factors Influencing Mental Health in Patients With Cancer

Social support is a crucial factor in the mental well-being of patients with cancer. The support of family, friends, and healthcare professionals can help patients cope with the emotional and psychological impact of cancer so that social support can be provided as emotional, informational, and practical support [36][37]. Emotional support is the most common form of social support and involves providing comfort, empathy, and encouragement to patients [38]. It helps patients feel less isolated, as well as manage their emotions and reduce stress levels. Therefore, family and friends can provide emotional support by listening to patients and their concerns [39].Informational support is another important form of social support [40]. Patients with cancer often have many questions about their diagnosis, treatment options, and prognosis. Healthcare professionals can provide patients with accurate and reliable information to help them make informed decisions about their care [41][42].

Patients with cancer may need help with daily tasks such as cooking, cleaning, and transportation. Family and friends can provide practical support by helping with these tasks or by arranging for professional help if needed [43]. Hence, patients could focus on their treatment and recovery without worrying about the practical aspects of daily life.

One vulnerable subgroup comprises individuals with metastatic or advanced-stage cancers [44]. Indeed, patients in this group often experience severe psychological distress due to the uncertainty of their outcome and potential side effects of treatments on both physical and mental health [45]. Another vulnerable subgroup consists of pediatric patients with cancer [46]. Children and adolescents facing a cancer diagnosis navigate unique challenges, including disrupted education, social isolation, and uncertainties about their future development [47]. They may have difficulty expressing their emotions, leading to increased vulnerability to mental health disorders such as anxiety, depression, and PTSD [47]. Additionally, elderly patients with cancer constitute another vulnerable subgroup due to their specific physical, cognitive, and psychosocial characteristics [48]. Indeed, older individuals may already be facing age-related challenges, such as comorbidities, functional limitations, and social isolation [49]. Hence, cancer diagnosis exacerbates these vulnerabilities, leading to increased rates of depression and anxiety, as well as decreased QoL [49]. Furthermore, individuals from marginalized populations, such as racial and ethnic minorities, low-income communities, and those with limited access to healthcare, are vulnerable to mental health issues in the context of cancer [50].

## Importance of Integrated Supportive Services in Cancer Care

To provide comprehensive care and support to patients and their families, integrated supportive services are essential in cancer care. These services include counseling, social work, nutrition, and rehabilitation [51][52]. One of the key benefits of integrated support services is that they can help patients manage the physical symptoms of cancer and its treatment [53]. For example, patients may experience pain, fatigue, and other side effects of treatment that impact their QoL [54]. Integrated support services can provide interventions such as pain management, physical therapy, and nutrition counseling to help patients manage these symptoms and improve their overall well-being [55][56][57].

Cancer care can involve multiple healthcare providers and treatment modalities, which can be exhausting for both patients and their families. Finally, integrated support services can help patients and their families cope with cancer’s social and financial impact [58]. Cancer can significantly impact a patient’s ability to work and maintain financial stability, leading to stress and anxiety [59]. Also, social work and financial counseling can help patients and their families manage these issues and improve their overall well-being [60].

## Support Services for Mental Health and Well-being

Supportive Care Programs and Survivorship Services

Supportive care programs and survivorship services can play a critical role in addressing mental health issues and improving the overall well-being of cancer patients [53]. Indeed, it could provide various interventions (e.g., counseling, psychotherapy, and medication management) for patients with cancer and their families to manage mental health issues [61][62]. Therefore, these services could provide ongoing support and resources to help patients manage the emotional and psychological impacts (e.g., long-term effects of cancer treatment) of cancer [63]. Hence, healthcare professionals should prioritize the integration of supportive care programs and survivorship services in cancer care to ensure that patients receive the best possible care and support.

Psychotherapy and Counseling Opportunities for Patients With Cancer

Psychotherapy and counseling could improve the support of patients with cancer that they need to manage the emotional and psychological challenges associated with cancer [64]. Therefore, these interventions provide a safe and supportive environment for patients to explore their feelings and emotions related to their cancer diagnosis and treatment [65]. One of the key benefits of psychotherapy and counseling is the ability of patients to develop coping strategies, including relaxation techniques, mindfulness practices, and cognitive-behavioral therapy techniques [66][67]. Also, improving patients’ communication skills and strengthening their relationships with their families is another important benefit of psychotherapy and counseling [68]. Hence, proper psychotherapy and counseling could enhance the overall QoL of patients with cancer.

Peer Support Groups and Online Communities

Peer support groups and online communities can be valuable resources for patients with mental health issues [69]. These groups allow patients to share their experiences, learn from others, and gain new insights and perspectives on their mental health challenges [70][71][72]. Indeed, peer support groups and online communities can help patients reduce feelings of isolation and provide a sense of community and belonging [73].

Also, these groups provide patients with practical advice and tips for managing their mental health. Members of these groups can share information about coping strategies, self-care techniques, and other resources that can help patients improve their mental health and well-being [74][75].

Some important cancer support groups and online communities are listed as follows:

1. Cancer Support Community (CSC)

The CSC is a global network of support groups and online communities for individuals affected by cancer [76]. They offer a variety of programs, including support groups facilitated by licensed professionals, educational resources, and opportunities for networking and connecting with others who understand the challenges of living with cancer.

2. American Cancer Society’s (ACS) Cancer Survivors Network (CSN)

The ACS hosts the CSN, an online community that provides a supportive environment for cancer patients, survivors, and caregivers [77]. Members can participate in discussion boards, connect one-on-one with others, and find information on various cancer-related topics.

3. CancerCare Online Support Groups

CancerCare offers a selection of online support groups specifically designed for individuals affected by different types of cancer [78]. These groups, facilitated by professional oncology social workers, focus on creating a supportive space for sharing experiences, discussing concerns, and connecting with others facing similar challenges [78].

4. Stupid Cancer

Stupid Cancer is a global community and advocacy organization dedicated to young adult cancer patients, survivors, and caregivers [79]. Their online community provides a platform for young adults affected by cancer to connect, find resources, and access support through forums, social networking, and online events.

5. HealthUnlocked Cancer Community

HealthUnlocked hosts a diverse range of online communities, including a dedicated community for individuals affected by cancer [80]. The Cancer Community allows members to ask questions, share experiences, and support one another in a safe and inclusive environment.

Remember, support groups and online communities can provide emotional support, information, and a sense of belonging. However, they should not replace professional medical advice.

Importance of Addressing the Unique Needs of Caregivers

Cancer affects the patient and their families, particularly the caregivers who play a critical role in the patient’s care and recovery. Caregivers of patients with cancer often experience significant emotional, physical, and financial stress, which can have a negative impact on their own health and well-being [81]. Therefore, it is important to address the unique needs of caregivers of patients with cancer. Caregivers often experience various emotions, including anxiety, depression, and grief [82]. Also, practical support [83] is another essential need for caregivers of cancer patients. In other words, caregivers often have to handle multiple responsibilities, including managing medications, coordinating appointments, and providing emotional support to patients and their physical health [84]. Indeed, caregiving can be physically demanding, and they often neglect their own health needs. So, it is essential to encourage caregivers to prioritize their health by using regular check-ups, healthy diet, and exercises; consequently, the stress and burden of cancer are reduced, and the overall QoL for the patient and the caregiver improves [85][86].

## Barriers to Accessing Support Services

Stigma and Cultural Attitudes Towards Mental Health and Cancer

Stigma can lead to discrimination, social exclusion, and a lack of access to resources and support [87]. In the case of mental health, stigma is a major barrier to receiving appropriate treatment. Indeed, many individuals who experience mental health problems avoid seeking help due to fear of being judged or stigmatized [88]. Hence, delays in obtaining treatment could lead to negative consequences for the individual’s mental health and overall well-being [89]. Also, cancer is taboo in some cultures, and individuals may avoid discussing it and/or seeking treatment [90]. Indeed, some cultures may consider cancer as punishment or a sign of weakness, which leads to social exclusion and discrimination [90][91].

Hence, enhancing knowledge and promoting education about these conditions is important to address stigma and cultural attitudes towards mental health and cancer. Also, providing culturally sensitive care and support for individuals with cancer-related mental health problems is essential to create a more inclusive and supportive society [92].

Financial and Logistical Challenges in Accessing Supportive Services

Financial and logistical challenges are another of the most important barriers to accessing supportive services for individuals with cancer who are also experiencing mental health problems [93]. Indeed, the cost of cancer treatment (e.g., medications) can be high, and many individuals may be faced with the additional costs (e.g., for receiving support services) associated with mental health care [94][95].

In addition to financial challenges, logistical challenges can make it difficult for individuals with cancer to access mental health support services [96]. Many individuals may live in rural or remote areas with limited access to mental health services. Others may have movement disorders or transportation challenges that make it difficult to access support services [97].

Role of Religion and Religious Beliefs On Preventive Receiving Supportive Services

For some communities, religion can be a source of comfort, strength, and hope, providing a framework for understanding and coping with the challenges of a cancer diagnosis [98]. This can promote resilience and psychological well-being; consequently reducing the likelihood of mental health issues [99]. Individuals who deeply integrate their religious beliefs into their daily lives may find solace in prayer, meditation, or seeking support from their religious community, which can serve as a form of emotional and spiritual support [100][101].

Also, religious beliefs can shape attitudes toward seeking professional mental health services by serving as both a facilitator and a barrier [102]. Some individuals may view mental health concerns as spiritual or moral struggles, believing that prayer, scripture study, or religious rituals alone can alleviate their distress [103]. In such cases, they may be less inclined to seek support from mental health professionals, which can hinder access to evidence-based interventions [104]. On the other hand, religious teachings may emphasize the importance of seeking help and provide a supportive framework for individuals to prioritize their mental health and well-being [105]. Religious leaders and organizations can play a crucial role in raising awareness about the importance of mental health services and reducing stigma within their communities [106].

Also, the cultural context could affect the availability and acceptability of supportive services. In some cultures, seeking external assistance for mental health concerns may be stigmatized or perceived as a lack of faith in religious healing [106][107]

Healthcare providers need to have cultural competence and an understanding of the influence of religious beliefs on patients’ attitudes toward mental health services [108]. Engaging in open and respectful conversations with patients about their religious beliefs can help them navigate potential conflicts or misunderstandings and identify appropriate means of support [109]. This may involve integrating religious practices into their care plan, considering the role of spiritual advisors, or collaborating with religious leaders to bridge the gap between religious beliefs and mental health services [109].

Strategies for Overcoming Barriers and Enhancing Service Accessibility

Several strategies can be implemented to overcome barriers and enhance service accessibility for mental health among patients with cancer. One of the most effective strategies is to increase awareness and education about the importance of mental health care for individuals with cancer [110]. This can be provided through public health campaigns, educational programs, and outreach efforts to healthcare providers and community organizations [111].

Another strategy is to provide integrated care that addresses both the physical and mental health needs of individuals with cancer [112]. This can involve collaboration between healthcare providers, mental health professionals, and other support services to ensure patients receive comprehensive care that addresses their needs [113].

Telehealth services [114] can also be an effective strategy for enhancing service accessibility for mental health among patients with cancer. Telehealth allows patients to access mental health services remotely, which can be particularly beneficial for individuals who live in rural or remote areas [115].

Also, financial assistance programs can help to overcome financial barriers to accessing mental health services [116]. These programs can provide financial support for therapy, medication, and other support services, making accessing their care needs easier for individuals with cancer [117].

Therefore, by addressing these challenges, we can help to ensure that individuals with cancer receive the comprehensive care and support they need to manage their conditions and improve their overall well-being.

## Future Directions and Recommendations

One important direction is to continue developing and implementing innovative mental health care approaches tailored to the unique needs of individuals with cancer [118]. For example, incorporating new technologies, such as virtual reality and artificial intelligence, into mental health interventions, as well as exploring new treatment modalities, such as mindfulness-based therapies and expressive arts therapies [119][120].

Also, it is essential to focus on improving access to mental health care for underserved populations, including individuals from low-income communities and racial and/or ethnic minorities [121]. It includes developing culturally sensitive interventions and support services, as well as addressing structural barriers to care, such as lack of insurance coverage and limited availability of mental health professionals in certain areas [122].

In addition, it is important to continue to prioritize research on the relationship between mental health and cancer, including the impact of mental health on cancer outcomes and the effectiveness of different mental health interventions for individuals with cancer [123].

Finally, it is necessary to continue to increase awareness about the importance of mental health care for individuals with cancer, both among healthcare providers and the general public [124]. This can involve developing public health campaigns and educational programs that highlight the importance of mental health care for individuals with cancer, as well as providing training and support for healthcare providers to ensure that they are equipped to meet the mental health needs of their patients [125].

## Conclusion

The impact of mental health among patients with cancer cannot be exaggerated. The emotional and psychological effects of a cancer diagnosis can be significant and can profoundly impact an individual’s overall well-being and QoL. Hence, it is essential to prioritize mental health care and support using various strategies for individuals with cancer that reduce these negative effects and improve patient outcomes.

## Conflict of Interests

All the authors declare there are no competing interests.
